# The ubiquity of phenotypic plasticity in plants: a synthesis

**DOI:** 10.1002/ece3.1603

**Published:** 2015-07-23

**Authors:** Kattia Palacio-López, Brian Beckage, Samuel Scheiner, Jane Molofsky

**Affiliations:** 1 Department of Plant Biology, University of VermontBurlington, Vermont, 05405; 2 Division of Environmental Biology, National Science Foundation4201 Wilson Blvd., Arlington, Virginia, 22230

**Keywords:** Adaptive plasticity, canalization, life-history traits, local adaptation, morphological traits, reciprocal transplant studies

## Abstract

Adaptation to heterogeneous environments can occur via phenotypic plasticity, but how often this occurs is unknown. Reciprocal transplant studies provide a rich dataset to address this issue in plant populations because they allow for a determination of the prevalence of plastic versus canalized responses. From 31 reciprocal transplant studies, we quantified the frequency of five possible evolutionary patterns: (1) canalized response–no differentiation: no plasticity, the mean phenotypes of the populations are not different; (2) canalized response–population differentiation: no plasticity, the mean phenotypes of the populations are different; (3) perfect adaptive plasticity: plastic responses with similar reaction norms between populations; (4) adaptive plasticity: plastic responses with parallel, but not congruent reaction norms between populations; and (5) nonadaptive plasticity: plastic responses with differences in the slope of the reaction norms. The analysis included 362 records: 50.8% life-history traits, 43.6% morphological traits, and 5.5% physiological traits. Across all traits, 52% of the trait records were not plastic, and either showed no difference in means across sites (17%) or differed among sites (83%). Among the 48% of trait records that showed some sort of plasticity, 49.4% showed perfect adaptive plasticity, 19.5% adaptive plasticity, and 31% nonadaptive plasticity. These results suggest that canalized responses are more common than adaptive plasticity as an evolutionary response to environmental heterogeneity.

## Introduction

Adaptation to environmental heterogeneity can occur in a variety of ways. Natural selection is expected to favor trait values that maximize fitness within a local environment (Linhart and Grant 1996; Anderson et al. 2014), but between environments, there are two possible evolutionary responses. Populations can differentiate genetically so as to become locally adapted (Futuyma and Moreno 1988; Kawecki and Ebert 2004; Gould et al. 2014) or individuals may be phenotypically plastic, expressing the optimal phenotype in both environments with no genetic differentiation (Bradshaw 1965; Schlichting 1986; Schlichting and Smith 2002).

Plasticity has been suggested as an adaptive mechanism that allows plants to optimally respond to environmental heterogeneity (Alpert and Simms 2002; Callahan et al. 2005). However, plasticity can be disfavored under a variety of circumstances, in some cases resulting in apparent maladaptive plasticity (Scheiner 2013). Nonadaptive plasticity can occur when a new environment induces a phenotype that is further away from the optimal phenotype (Ghalambor et al. 2007).

When the environment is spatially heterogeneous, local adaptation is expected if there is limited gene flow. However, when gene flow is extensive and there is a reliable environmental cue, phenotypic plasticity is favored (Emery 2009; Scheiner 2013). Extensive theoretical work has shown a broad ranges of conditions that favor or disfavor plasticity versus local adaptation (e.g., Levins 1963; Cohen 1968; Orzack 1985; Lynch and Gabriel 1987; Moran 1992; Gavrilets and Scheiner 1993; Sasaki and De Jong 1999; Tufto 2000; De Jong and Behera 2002; Sultan and Spencer 2002; Lande 2009; Scheiner 2013). However, we do not know how frequently such conditions are met. In the literature, it is frequently assumed that plasticity, especially adaptive plasticity, is very common (Schlichting 1986; Agrawal 2001; Sultan and Spencer 2002; Crispo et al. 2010; Nicotra et al. 2010; Valladares et al. 2014). Two prior studies (Leimu and Fischer 2008; Hereford 2009) examined the prevalence of local adaptation but focused exclusively on traits closely related to fitness. For example, Leimu and Fischer (2008) examined the evidence from reciprocal transplant studies and reported local adaptation in 45% of 35 plant studies. In the remaining 55% of the cases that did not show local adaptation, Leimu and Fischer did not specifically address the type and prevalence of phenotypic plasticity. In a study that included both animals and plants, Hereford (2009) found evidence of local adaptation in 71% of reciprocal transplant studies but also did not classify the type and prevalence of phenotypic plasticity. Moreover, these studies were limited to traits related to fitness so could not address if some traits were more likely to be locally adapted and other traits within the same species were phenotypically plastic.

Our analysis differs from earlier studies that used data from reciprocal transplants to focus exclusively on the question of local adaptation and fitness. In contrast, in this study, we use the data from such studies to address the prevalence and type of phenotypic plasticity for all possible traits (morphological, physiological, and life history). Secondarily, we also address how often that plasticity appears to be adaptive. Our secondary question requires an assumption about whether populations are adapted to their resident habitats, an issue we return to in the Discussion. We confine our analysis to reciprocal transplant studies on plants because plants are sessile and the physical environment at a local spatial scale directly determines their survival and growth. Reciprocal transplant experiments allow us to identify whether phenotypic differences among sites are due to environmental effects or genetic differentiation (McGraw and Antonovics 1983; Ghalambor et al. 2007). Yet in plants, the high degree of spatial variation that can occur at local scales (Linhart and Grant 1996) sets the stage for plasticity to be an important mechanism of adaptation to fine-scale environmental heterogeneity.

**Figure 1:**
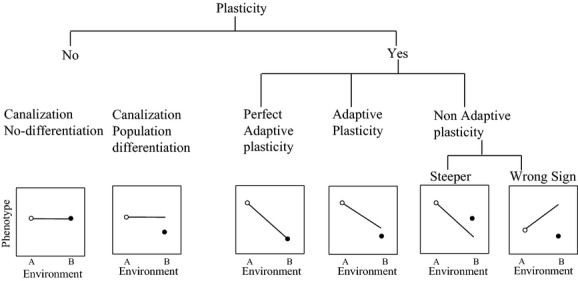
Five possible evolutionary responses to the environment: (1) canalized response–no differentiation; (2) canalized response–population differentiation; (3) perfect adaptive plasticity: plastic, reaction norms not different; (4) adaptive plasticity: plastic reaction norms with the same slope but different intercepts; and (5) nonadaptive plasticity: plastic reaction norms that are steeper than the optimum or the slope of the reaction norm is in the opposite direction than the optimal reaction norm. Circles indicated the optimal phenotype for population A (open circle) and population B (closed circles). The figure only shows the reaction norm for population A. The end of the line shows the mean phenotype of population A growing in environment B, the foreign environment.

We categorize five possible evolutionary patterns based on the traits of a population in its resident environment and in its nonresident environment relative to the other population in that nonresident environment. First, canalized response–no differentiation refers to the situation in which there are no plastic responses between the two environments and also the means are not different. Thus, the phenotype in the resident environment is the same as that in the nonresident environment and the same for both populations (Fig. 1). Second, canalized response–population differentiation refers to the conditions in which neither population is plastic between the two environments; in addition, the mean phenotypes of the two populations are different (Fig. 1). Next, we categorize three types of phenotypic plasticity following Ghalambor et al. (2007). First, perfect adaptive plasticity refers to the conditions in which there are different phenotypic responses between the environments but the reaction norms between the two environments are not different. In this case, both populations exhibit phenotypic plasticity with the nonresident population exhibiting a similar or the same phenotype as the resident population (Fig. 1). Second, adaptive plasticity refers to when the resident population and the population that is nonresident respond in a similar way to the environment resulting in parallel but not congruent reaction norms; thus, the phenotypic expression of the nonresident population does not match the phenotypic expression of the resident population. Third, nonadaptive plasticity refers to the situation in which both populations are plastic across the two environments, but the slopes of the reaction norms are different (Fig. 1). In this last case, the mismatch in slopes that we refer to as nonadaptive plasticity can occur in two different ways, steeper and wrong sign. First, reaction norms can be steeper than the optimum reaction norm (Fig. 1), and it may occur when the phenotypic expression in the nonresident environment is in the correct direction but overshoots the optimal expression. Second, wrong-sign non adaptive plasticty (Fig.1) occurs when the slope of the reaction norm is in the opposite direction than the optimal reaction norm. We recognize that our analysis focuses on among-site environmental heterogeneity and does not address possible patterns of adaptation to within-site or micro-environmental heterogeneity. However, we have no reason to expect that the general patterns found among sites should differ within sites.

Our central question is addressed by calculating the relative number of traits that fit the nonplastic evolutionary scenarios versus those that fit the plastic ones. The secondary question is addressed by partitioning the total traits analyzed among the five evolutionary responses to the environment (canalized response–no population differentiation, canalized response–population differentiation, perfect adaptive plasticity, adaptive plasticity, and nonadaptive plasticity). Thus, our framework allows us to simultaneously evaluate in a synthetic framework different evolutionary responses to the environment.

## Methods

### Data collection

We searched for published papers from the ISI *Web of Science* using the keywords “local adaptation,” “reciprocal transplant,” and “adaptive evolution.” We also looked for papers included in similar meta-analyses (e.g., Leimu and Fischer 2008; Hereford 2009). Most of these studies were not focused on the evolution of phenotypic plasticity and, thus, more likely to be representative of patterns of plasticity. Studies involving newly invasive species were not included (see Discussion).

In contrast to other meta-analysis (e.g., Leimu and Fischer 2008; Hereford 2009), we only used reciprocal transplant studies (i.e., at least two populations grown in their resident and a nonresident environment). Thus, we excluded studies that used a common garden approach or measured plasticity in the greenhouse or field plots. In addition, each of our chosen studies had to measure at least 10 individuals from each population and to have reported a measure of intrapopulation variation (i.e., variance, standard deviation, standard error). For multiyear studies, we used the data only from the first year for consistency among the studies. For each of the studies selected, we recorded the mean of each trait, its variation, and sample size. We represent these reciprocal transplant experiments using the following notation: A in A (“*AinA*”) represents population A grown in its resident environment A, A in B (“*AinB*”) represents population A growing in the nonresident environment B, B in B (“*BinB*”) represents population B growing in its resident environment, and B in A (“*BinA*”) represents population B growing in the nonresident environment of population A (Fig. 1).

### Data analyses

We subsequently analyzed our dataset in two different ways: by “paired” record and then by “blocked” record. We considered a paired population record to consist of a population grown in both its resident and nonresident environment, for example, both *AinA* and *AinB*, and *BinB* and *BinA* are pairs. Here, we analyze each population within a study independently from the other population for each trait. We considered a blocked record to consist of the pair of pairs, for example, *AinA, AinB, BinB,* and *BinA* for a given study. Here, we analyze both populations within study together for each trait. We estimate the prevalence of plasticity using both methods of analysis, for example, by paired and by blocked records. We further decompose plasticity into five subcategories, for example, canalized response and no differentiation, canalized response and population differentiation, perfect adaptive plasticity, adaptive plasticity, and nonadaptive plasticity. We first estimated the prevalence of plasticity in each population by computing the standardized difference between trait values in resident (record value reported as *AinA* or *BinB*) and nonresident environments (record value reported as *AinB* or *BinA*):


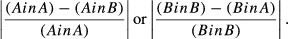
(1)

where this metric is a proxy for phenotypic plasticity. The metric ranges from 0 to infinity with values near 0 indicating a lack of plasticity and values away from 0 are indicative of plasticity. We also calculated the prevalence of plasticity using equation 1 but using the block analysis; here, the equation has the additional condition that both paired populations had to be classified as plastic for a block to be considered plastic, that is, the same trait for both populations had to be scored above the threshold to be categorized as being plastic. The fractional estimates of plasticity using paired and blocked analyses were normalized using different subsets of the records, so that we do not necessarily expect that the block estimates of plasticity should be less than the paired estimates.

We choose a threshold effect size of 0.53 to categorize records as plastic or nonplastic as well as to distinguish other categorizations as noted below. This threshold was based on the mean CV (coefficient of variation) calculated across all traits and studies. This effect size is equivalent to one standard deviation, a difference that would be statistically significant at *P* < 0.05 for a sample size of 10, our minimum sample size. We report the fraction of records displaying phenotypic plasticity based on this threshold, but recognize that this threshold is somewhat arbitrary. We therefore also performed a sensitivity analysis where both doubled and halved threshold value was used to assess resultant changes in our results. Furthermore, we also calculate the CDF (cumulative distribution function), which represents the fraction of records within a given threshold value, and thus is a measure of the fraction of a population within a given effect size. The inclusion of the CDF plot for this metric and the others that follow allows the reader to choose their own threshold value.

We subclassified records categorized as nonplastic into two categories, canalized response–no population differentiation and canalized response–population differentiation, using blocked records (Fig. 1). We define blocks as being a canalized response–no population differentiation based on a lack of difference across populations and environments, whereas a canalized response–population differentiation is characterized based on trait differences at the threshold of 0.53. Our assessment was based on the following metric:


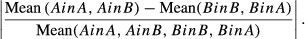
(2)

This metric varies on the range 0 to infinity, with values near 0 indicating no difference in traits across populations from two environments (i.e., canalized response–no population differentiation), while values away from 0 are representative of different trait values (i.e., canalized response–population differentiation). We used the 0.53 threshold to distinguish between these cases.

We subcategorized population trait records that were classified as plastic based on analysis of paired records. We estimated the difference between the trait value in the resident and nonresident environments, standardized by the difference in the resident populations grown in each environment:


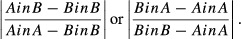
(3)

This metric varies on the range 0 to infinity, with values from 0 to our 0.53 threshold representing perfect adaptive plasticity (cases where the nonresident population had trait values that closely matched those of the resident population when both were grown in the environment of the resident population). Values <1 but >0.53 represent adaptive plasticity (cases where the trait values of nonresident populations moved closer to the resident trait values, but were less close than those classified as perfectly adaptively plastic). Finally, values >1 represent nonadaptive plasticity (cases where the trait values of nonresident populations diverged from resident populations when grown in the environment of the resident population). We furthermore characterized nonadaptive plasticity into reaction norms that are “too steep” resulting in an overshooting of the optimal trait value, which was identified by the following condition, for example, for population A: (*AinA*>*BinB* and *AinB*<*BinB*) or (*AinA*<*BinB* and *AinB*>*BinB*). “Wrong-sign” nonadaptive plasticity occurs when the slope of the reaction norm is in an opposite direction to that of the optimal reaction norm, for example, identified when (*AinA*>*BinB* and *AinB*>*AinA*) or (*AinA*<*BinB* and *AinB*<*AinA*).

We also categorized plasticity based on the difference between the trait values of paired populations grown in two environments using blocked records. In this metric, we choose the larger of the two differences, and standardizing by the difference in mean trait values grown in each environment:


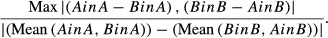
(4)

This metric again varies on the range 0 to infinity and is interpreted similarly to eq. 3, with values near 0 representing perfect adaptive plasticity and values away from 0 represent either adaptive or nonadaptive plasticity. The use of both equations 3 and 4 provides for an additional measure of the robustness of our results.

We bootstrapped confidence intervals for the CDFs using 5000 resampled datasets and three resampling methods. The first method was to resample the original records with replacement. Each resampled record consisted of a set of all four trait values (*AinA, AinB, BinB, BinA*), which were resampled as a single unit. In the second method, we resampled the sets as above but then also generated a new value for each member of the set using the standard error of the mean for each trait, calculated from the reported mean and standard error of a record, and assuming a correlation of 0 between each member of the set. In the third method, we set the correlation among the random deviates to be 1, so that the random deviates of each component of a set were perfectly correlated. The contrasting assumptions of correlations of 0 and 1 among random deviates allow us to bracket the range of likely correlations among populations, assuming that correlations were non-negative. All analyses were carried out in R, ver. 3.1.2 (R Core Team, 2013); the code is available from the authors upon request.

## Results

We found 31 studies that met our criteria (Table S1). The studies included 15 plant families, representing different life histories (herbaceous annual and perennials, grasses, and shrubs) and nine different environments. Of the 31 studies, four were on shrubs, four were on grasses, and the remainder were on herbaceous plants and these were split equally among annual and perennial plant species (Table S1). The data consisted of 181 records (individual traits) and the number of traits per study ranged from 1 to 14 with a median of three traits, (Table S1). All traits measured in a study were included in the analyses: 50.8% of the records were life-history traits, 43.6% were morphological traits, and 5.5% were physiological traits. By including all measured traits, we reduced possible selection bias by the investigator.

**Figure 2:**
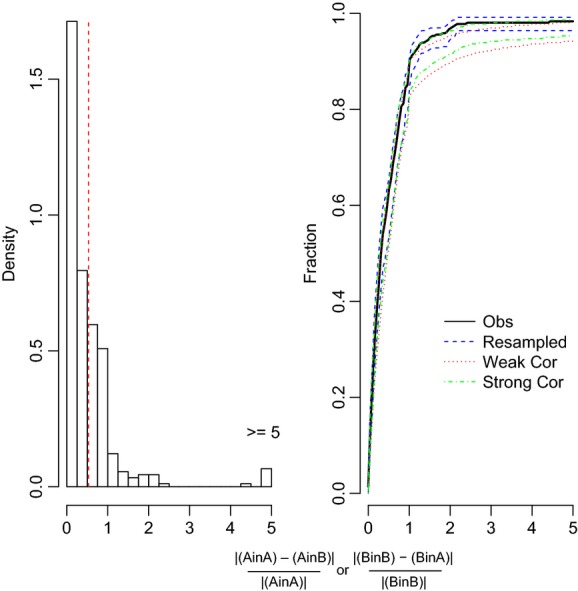
Histogram and cumulative distribution function for population trait pairs indicating plastic versus not plastic. Population trait pairs with values below the threshold (0.53, indicated by the dashed vertical line in the histogram) for both traits were categorized as not plastic.

We found that nearly two-thirds (64.1%) of the trait records showed no plasticity when analyzed by population pairs (Fig. 2; Table 1A), and over half (51.9%) were not plastic when analyzed by block (Table 1B). Our sensitivity analyses showed that a large proportion of the records showed no plasticity even when we shifted the threshold to half its value (i.e., 0.265), with 44.2% of the records being nonplastic by pair and 33.7% nonplastic by block (Table S2). On the other hand, if the threshold was doubled (1.06), the majority of the records were nonplastic with 91.2% by pair and 83.4% by block (Table S3). We bootstrap the data to illustrate the uncertainty in our results (Figs. 5).

**Table 1:** (A) The relative frequencies of plastic versus nonplastic traits based on comparing trait values of a pair of sets of individuals from a single population grown in two locations. (B) The relative frequencies of the five patterns based on comparing trait values of a block of four sets of individuals from two populations grown in two locations. All categorization was based on a cumulative distribution function threshold of 0.53 (Figs. 5)

(A) Records by pair	*N*	Not plastic	Plastic
	362	64.1	35.9

(B) Records by block	*N*	Not plastic	Plastic
	181	51.9	48.1

					Adaptive plasticity		
	N	Pattern 1: Canalization no differentiation	Pattern 2: Canalization population differentiation	Pattern 3: Perfect Adaptive plasticity	Pattern 4: Adaptive plasticity	Pattern 5: Non adaptive plasticity
All	181	8.8	43.1	23.8	9.4	14.9
Life history	92	8.7	39.1	23.4	10.9	17.9
Morphological	79	6.3	45.6	26.6	7.6	13.9
Physiological	10	30	60	10	0	0

**Figure 3:**
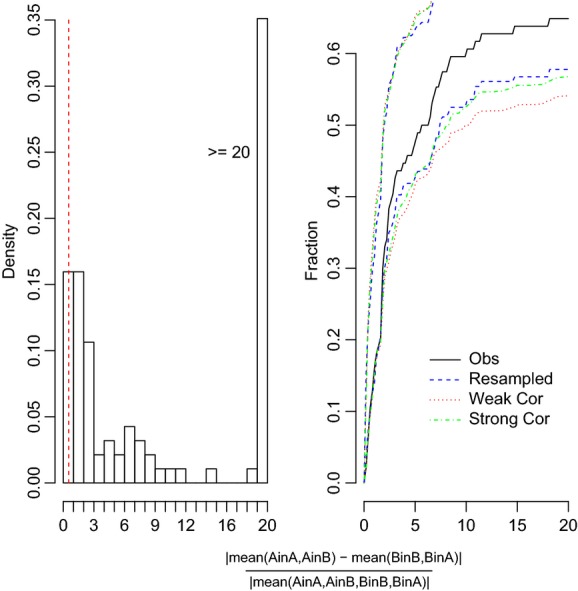
Histogram and cumulative distribution function for nonplastic trait pairs with canalized response–no differentiation versus canalized response–population differentiation. Population trait pairs with values below the threshold (0.53, indicated by the dashed vertical line in the histogram) were categorized as canalized response–no differentiation.

**Figure 4:**
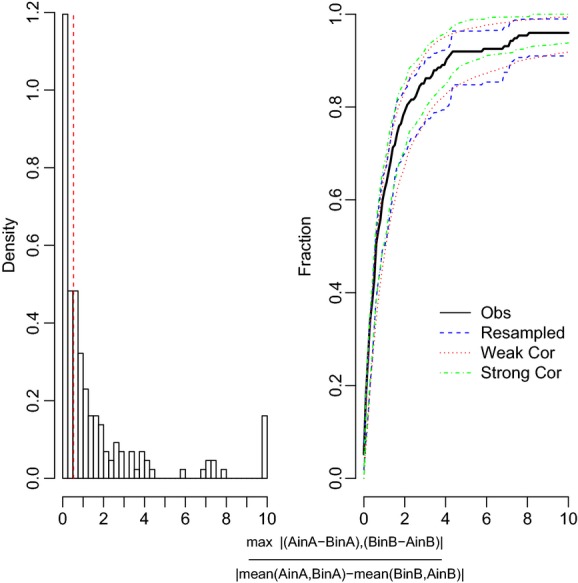
Histogram and cumulative distribution function for population trait pairs with perfect adaptive plasticity versus adaptive plasticity or nonadaptive plasticity. Population trait pairs with values below the threshold (0.53, indicated by the dashed vertical line in the histogram) were categorized as being perfect adaptive plastic.

**Figure 5:**
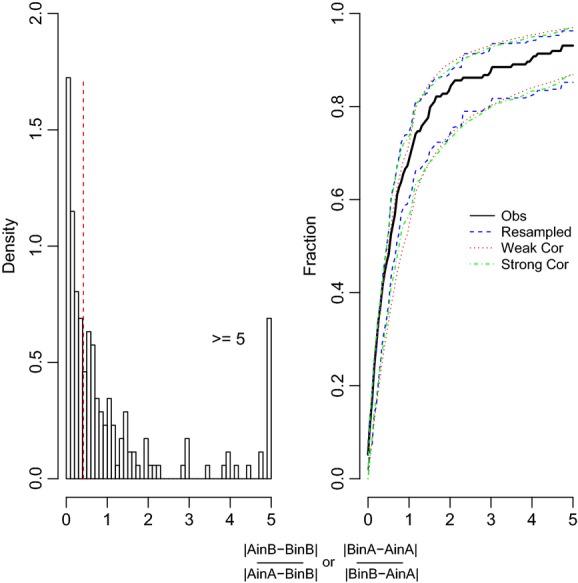
Histogram and cumulative distribution function for trait pairs with plasticity. Trait pairs with values from 0 to 0.53 represent perfect adaptive plasticity, values <1 but >0.53 represent adaptive plasticity, and values >1 represent nonadaptive plasticity.

In the block analyses and using eq. 2 for those traits records that showed no plasticity, only a small subset of our nonplastic records was canalized response–no population differentiation (8.8%), meaning that there was no local differentiation (Fig. 3), but the majority (43.1%) showed trait differences between the population pairs (Fig. 3, Table 1). The remaining trait records (48.1%) were plastic. If we consider only the plastic traits by block and apply eq. 3, then we found that 49.4% of the total records showed perfect adaptive plasticity (Table 1), 19.5% indicated adaptive plastic and 31% showed nonadaptive plasticity (Figs. 5; Table 1). Of traits that showed nonadaptive plasticity, 31.5% had steeper reaction norms and 68.5% had wrong-sign reaction norms. The percentage of perfect adaptive plasticity was consistent for the two equations we used, 49.4% when using eq. 3 and 44.3% when using eq. 4 (Fig. 4). When we doubled or halved our threshold, the proportion of plastic traits changed to 16.6% (Table S3) or 66.3%, respectively, primarily due to substantial increases in the number of records classified as having different reaction norms (adaptive or nonadaptive plasticity; Table S2). The percentage of records classified as having different slope reaction norms (nonadaptive plasticity) was similar for the three thresholds (0.53, 0.265, and 1.06) at 31%, 30.8%, and 25%, respectively.

When partitioned by type of trait, life-history and morphological traits showed similar patterns to each other and to the overall pattern (Table 1). Physiological traits differed in their pattern, but their sample size was substantially smaller and thus, too small to draw firm conclusions. The similarity between life-history and morphological traits persisted when we changed the thresholds (Tables S2 and S3).

The individual studies varied in the number of traits measured. If the traits were highly correlated then we would expect all of the traits in a given study to be scored with the same pattern, indicating that our estimate of the frequency of the various patterns might be biased. To address this partial bias, we examined whether the same pattern was clustered within studies. We found no such tendency (Fig. S1).

## Discussion

Phenotypic plasticity is assumed to commonly occur in plant populations (Schlichting 1986; Dudley and Schmitt 1996; Franks et al. 2014; Merila and Hendry 2014) but has been hypothesized to differ between fitness and nonfitness-related traits (Sultan 2000). However, in our study across all traits, we found that plasticity was not as common as nonplastic responses. When we examine plasticity in only traits related to fitness, we also find that plasticity was not as common as canalization. Fitness-related traits should have reduced plasticity because they are under stronger selection (Kingsolver et al. 2012). However, the life-history traits that we included in our analysis are fitness components, rather than absolute measures of an individual's fitness. It may be that trade-offs in the plasticity expressed among fitness components result in overall lower levels of plasticity for fitness itself. Unfortunately, these data do not permit an analysis of trade-offs among traits because trait correlations were rarely reported; this question remains for future studies.

Our analyses were predicated on a key assumption. We assumed that each population in a reciprocal transplant experiment was optimally adapted to its resident environment, and that the trait expression of the resident population in its own environment measured the optimal phenotype in that environment. We emphasize that the above assumption does not affect the answer to our core question (i.e., the prevalence of phenotypic plasticity), but is necessary to address our secondary question (i.e., how often plasticity appears to be adaptive). Ideally, we would want to know the relationship between each trait and its effect on fitness, but such data are not available. Instead, we assumed that each population is currently at its evolutionary equilibrium in its resident environment and thus has achieved an optimal phenotype in this location. Because our analyses required this assumption, we excluded studies of any newly invasive species that were unlikely to be at this evolutionary equilibrium.

Many studies have documented adaptive phenotypic plasticity (DeWitt and Scheiner 2004). For example, in a study on 13 populations of cork oak *Quercus suber*, plasticity for specific leaf area and leaf size was associated with an adaptive advantage for dealing with variable temperature and rainfall regimes (Ramírez-Valiente et al. 2010). Similarly, Anderson et al. (2012) found plasticity in flowering time in the species *Boechera stricta* (Brassicaceae) in response to temperature. In this case, phenotypically plastic genotypes were able to accelerate flowering time which resulted in a fitness advantage. A recent review by Franks et al. (2014) tested how frequently evolution or plastic responses occur in response to climate change and whether these two strategies co-occur. The majority of studies showed that both genetic and plastic responses are occurring in response to climate change and that these two strategies are not mutually exclusive. However, that analysis did not separate how much of the adaptive responses in each case were due to genetic or plastic changes in individual traits. Along with other studies, our study provides a framework for comparing the relative frequencies of adaptive plasticity and local adaptation or canalized responses. This comparison is important because models of plasticity evolution make predictions about the relative frequencies of these evolutionary outcomes (e.g., Chevin and Lande 2010; Scheiner 2013), and adaptive plasticity is often assumed to commonly occur (Chevin et al. 2010; Valladares et al. 2014).

If we define beneficial plasticity as plasticity that increases mean fitness across environments (in our case, those traits showing perfect adaptive plasticity), then nonplastic modes of adaptation (canalized response–no population differentiation + canalized response–population differentiation) are the more common evolutionary strategy (perfect adaptive plasticity = 23.8% vs. all nonplastic outcomes = 51.9%). If we conservatively define beneficial plasticity to include both the perfect adaptive and adaptive plasticity classes, then beneficial plasticity still represents only 33.2% of the total trait records, again less than all nonplastic adaptation. Therefore, our analyses lead to the conclusion that adaptive plasticity is less common than canalization. This conclusion is robust to our assumption that populations are locally adapted because our conclusions are not predicated on showing that the empirical studies showing nonplasticity are in fact locally adapted.

For plastic traits, our conclusions about the frequency of perfect adaptive plasticity represent an upper bound. If trait values of the resident populations do not represent the local optimum, then trait pairs categorized as having the same reaction norm (perfect adaptive) are not actually perfect. For population trait pairs categorized as having reaction norms with same slope and different intercepts, even if one of the pair is actually the optimal or perfect reaction norm, the other cannot be, so our designation of “suboptimal” is still correct for that population trait pair. For population trait pairs having reaction norms with different slopes, if one is optimal the other has to be maladaptive. Thus, if our assumption is incorrect it would bias our results toward overestimating the frequency of beneficial plasticity, making perfect adaptive plasticity even less common than assumed.

Our analyses required us to make assumptions concerning the numerical value of the threshold for deciding when a trait fell within a given pattern. We had to choose some threshold and the trait value distributions do not show any obvious breakpoint (Figs. 5). A sample size of 10 was the minimum sample size for inclusion in our analyses, so this threshold is conservative in categorizing means, elevations or slopes as different. In addition, the bootstrapping of the CDF takes into account the uncertainty of our results; moreover, we can choose different breakpoints and see how our assumptions alter our interpretation of the plasticity patterns as we did when we double or half our threshold value (Tables S2 and S3). Because the CDF was based on the pooled data and we are asking about the relative frequency of different categories; setting a threshold is similar to the process of interpreting the effects of a pooled effect size in a standard meta-analysis. The difference is that a standard meta-analysis is typically framed as a hypothesis test (e.g., Does treatment X differs by treatment Y across a set of studies? Gurevitch and Hedges 2001) rather than as an analysis of relative frequencies.

One check of our categorization is to compare it with those of Leimu and Fischer (2008) and Hereford (2009) for those traits that were common among the studies, 29 for the former and 20 for the latter. Unfortunately, such a comparison cannot be carried out because of different criteria and assumptions. Both of these other studies examined traits that the authors categorized as fitness and assumed that greater values always represented higher fitness under the assumption that fitness is always under directional selection. In contrast, we assumed that even life-history traits are just fitness components that may be under stabilizing selection. Both of the other studies used a different metric than we used. They categorized local adaptation by comparing the trait value of the resident population growing in the resident environment with that of the nonresident population growing in that same environment (compare with our eq. 1).

One surprising result from our analyses is the relatively high frequency of nonadaptive plasticity across all traits. Yet, apparent maladaptive plasticity may not actually be so. Recent simulation models identified two conditions under which selection might result in reactions that deviate from the optimum. In both instances, selection is on bet-hedging rather than on plasticity per se. Scheiner and Holt (2012) found that hyperplasticity – a reaction norm much greater than optimal – could be selected for as a form of bet-hedging when the environment is highly heterogeneous and the environmental cue is unreliable. Scheiner (2014) found that if developmental instability is pleiotropic with plasticity, then selection for instability as a form of bet-hedging could result in maladaptive plasticity. Genetic correlations between trait plasticity and either trait means or plasticities of other traits also could be responsible for nonadaptation. This last explanation is unsatisfying in that it attributes nonadaptation to unmeasured effects. More information on the quantitative and molecular genetics of plasticity is needed.

Under ideal conditions, we expect plasticity to be favored over local adaptation any time that individuals or lineages experience heterogeneous environments due to either temporal variability or spatial heterogeneity coupled with movement (Lloyd 1984; Lively 1986; Sultan 1987; Schlichting and Levin 1990). Thus, although the magnitude and pattern of plasticity can vary among organisms, traits, and environments, plasticity is considered as a ubiquitous and common mechanism in nature (Murren et al. 2014). Yet, we found that adaptive plasticity was the less frequent outcome. This may indicate that local populations experience environmental heterogeneity less often than we might expect, or that other factors are inhibiting selection for plasticity. We find the first possibility unlikely, especially for plants, although it may be that the extent of environmental differences between the reciprocal transplant gardens was outside the range of environmental heterogeneity normally experienced within each population (Ghalambor et al. 2007). But that would not explain a lack of plasticity, as none of these populations came from strictly uniform environments.

Many factors can inhibit selection for plasticity, including various costs and limitations (DeWitt et al. 1998; Scheiner et al. 2012; Scheiner 2013, 2014). However, there is little empirical evidence about the relative importance of those various factors. For some, such as costs of plasticity, the data are mixed (e.g., Scheiner and Berrigan 1998; Van Kleunen et al. 2000; Weinig et al. 2006; Steiner and van Buskirk 2008; Aubret and Shine 2010). For others, such as links with developmental instability, the lack is due to technical difficulties of measurement (e.g., Tonsor et al. 2013). Lastly, for those such as cue reliability, the lack is mostly due to a failure to measure the relevant ecological and life-history parameters. As theory now points to which conditions are more likely to favor plasticity or local adaptation, focused empirical studies can answer the question raised by our analysis: Why is local adaptation/canalization more common than adaptive plasticity?

## Supporting Information

**Figure S1.** Distribution of the different patterns of plasticity of the traits within each study.

**Table S1.** Summary of the reciprocal transplant studies included in the analyses. **Table S2.** (A) The relative frequencies of plastic versus nonplastic traits based on comparing trait values of a pair of sets of individuals from a single population grown in two locations. (B) The relative frequencies of the five patterns based on comparing trait values of a block of four sets of individuals from two populations grown in two locations. **Table S3.** (A) The relative frequencies of plastic versus nonplastic traits based on comparing trait values of a pair of sets of individuals from a single population grown in two locations. (B) The relative frequencies of the five patterns based on comparing trait values of a block of four sets of individuals from two populations grown in two locations.
