# Supplementary figures and images for: The ubiquity of phenotypic plasticity in plants: a synthesis
Source: Ecol Evol. 2015 Jul 23;5(16):3389–400. doi: 10.1002/ece3.1603 (PMC4569034; doi:10.1002/ece3.1603)

fig. S1

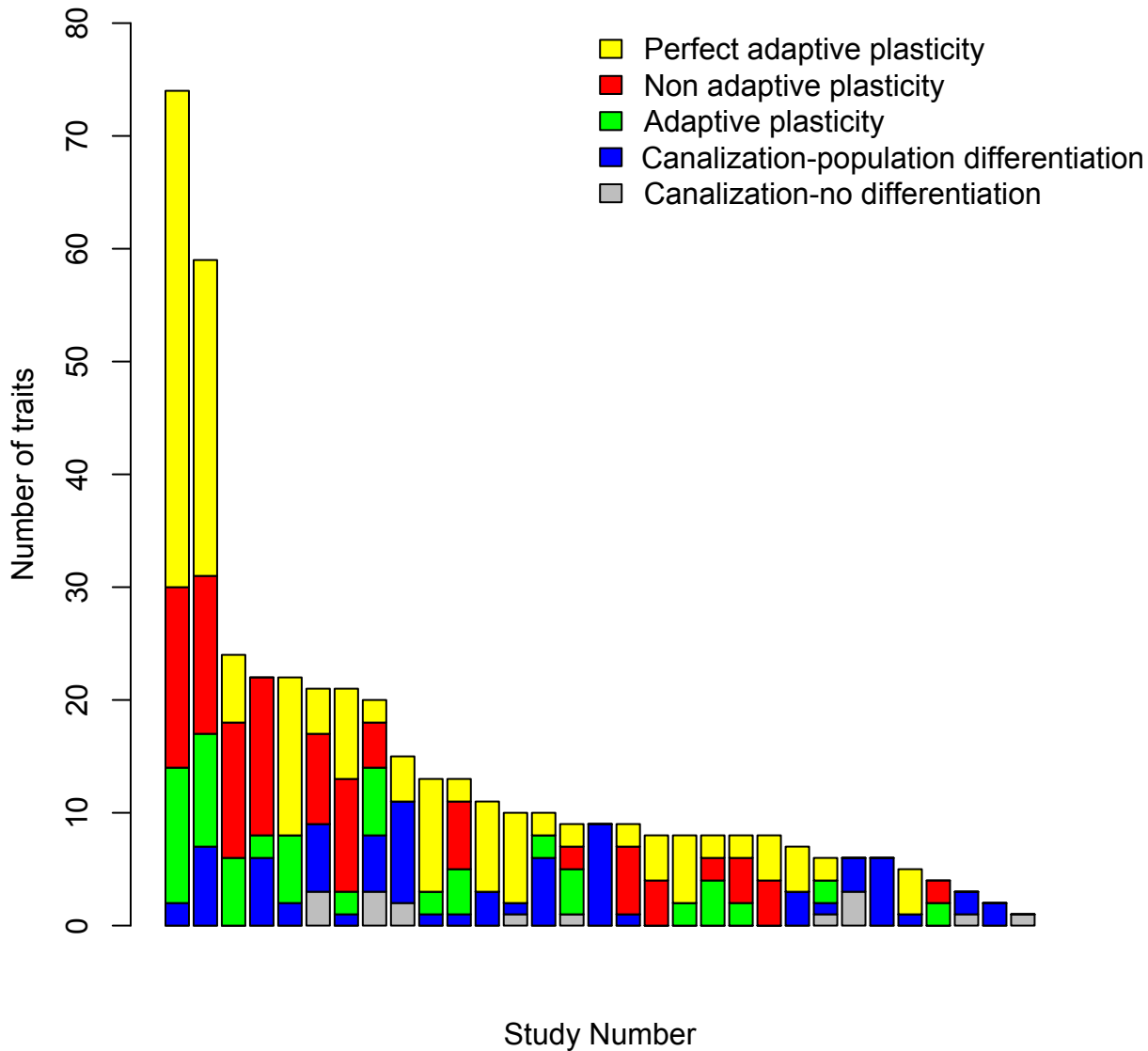

Supplement: Supplementary file 1 — Figure S1. Distribution of the different patterns of plasticity of the traits within each study. [file ece30005-3389-sd1.pdf]
