# Supplementary material for: The ubiquity of phenotypic plasticity in plants: a synthesis
Source: Ecol Evol. 2015 Jul 23;5(16):3389–400. doi: 10.1002/ece3.1603 (PMC4569034; doi:10.1002/ece3.1603)
Supplement: Supplementary file 2 [file ece30005-3389-sd2.docx]

**Appendices**

**Table S1**

| **Reference** | **Trait Categories** | **Trait** | **Growth form** | **Family** | **Life form** | **Environment** |
| --- | --- | --- | --- | --- | --- | --- |
| Anderson and Geber (2010) | M | Relative growth rate | Shrub | Ericaceae | Perennial | Deciduous forest |
| Bennington and McGraw (1995) | M | Leaf area | Herb | Balsaminaceae | Annual | Deciduous forest |
|  | LH | CH/CL No. of seed |  |  |  |  |
|  | LH | Mean no. CH seed/capsule |  |  |  |  |
|  | LH | Fitness |  |  |  |  |
| Callahan and Pigliucci (2002) | M | Leaf number | Herb | Brassicaceae | Annual | Old field |
|  | LH | Bolting day |  |  |  |  |
|  | LH | Rossette diameter |  |  |  |  |
|  | LH | Influorescence height |  |  |  |  |
|  | LH | No. of fruits |  |  |  |  |
| Chapin and Chapin (1981) | M | Tiller height | Herb | Cyperaceae | Perennial | Alpine tundra |
|  | M | Number of leaves |  |  |  |  |
| Donohue et al. (2000) | LH | Fitness (No. Seeds) | Herb | Balsaminaceae | Annual | Old field |
|  | LH | Relative fitness(LS means) |  |  |  |  |
| Donohue et al. (2001) | M | Internode 1 | Herb | Balsaminaceae | Annual | Old field |
|  | M | Internode 2 |  |  |  |  |
|  | M | Height |  |  |  |  |
|  | M | No. of nodes |  |  |  |  |
|  | LH | Primary flowers |  |  |  |  |
|  | M | Primary branches |  |  |  |  |
|  | LH | Quiescent buds |  |  |  |  |
|  | M | Leaf length |  |  |  |  |
|  | LH | Flowering date |  |  |  |  |
|  | LH | Fitness (No. seeds) |  |  |  |  |
|  | LH | Relative fitness |  |  |  |  |
| Emms et al. (1997) | M | Leaf area | Herb | Iridaceae | Perennial | Wetland |
|  | LH | Survival |  |  |  |  |
|  | M | New ramets per rizhome |  |  |  |  |
| Etterson (2004) | LH | Log fecundity | Herb | Fabaceae | Annual | Desert |
|  | M | Log leaf number |  |  |  |  |
|  | M | Log specific leaf area |  |  |  |  |
|  | LH | Reproductive stage |  |  |  |  |
| Fornoni et al. (2003) | M | Plant size (leaf number) | Herb | Solanaceae | Annual | Tropical dry forest |
|  | LH | Fitness |  |  |  |  |
|  | M | Resistance |  |  |  |  |
| Fritsche and Kaltz (2000) | M | Number of ramets | Herb | Lamiaceae | Perennial | Old field |
|  | LH | Vegetative reproduction |  |  |  |  |
|  | LH | Proportion surviving plants |  |  |  |  |
|  | LH | Proportion of flowering plants |  |  |  |  |
| Godoy et al. (2011) | M | Height | Herb | Lamiaceae | Perennial | Temperate rainforest |
|  | M | Change in height |  |  |  |  |
|  | M | Cover area |  |  |  |  |
|  | M | Change in cover area |  |  |  |  |
|  | M | Leaf number |  |  |  |  |
|  | M | Change in No. of leaves/month |  |  |  |  |
|  | LH | No.of inflorescences/individual |  |  |  |  |
| Griffith and Watson (2005) | LH | Pre flowering branches | Herb | Asteraceae | Annual | Old field |
|  | LH | Senescence time |  |  |  |  |
|  | LH | Flowering time |  |  |  |  |
| Hall and Willis (2006) | LH | Corolla width | Herb | Scrophulariaceae | † | Coastal |
|  | LH | Corolla tube length |  |  |  |  |
|  | M | Leaf width |  |  |  |  |
|  | M | Stem thickness |  |  |  |  |
|  | LH | Days to flowering |  |  |  |  |
|  | M | Leaves produced |  |  |  |  |
|  | M | Maximum height |  |  |  |  |
|  | LH | Maximum rossette diameter |  |  |  |  |
|  | LH | Survival to flowering |  |  |  |  |
|  | LH | Flowers per plant |  |  |  |  |
|  | LH | Seeds per flower |  |  |  |  |
|  | LH | Flower/plant (flowering only) |  |  |  |  |
|  | LH | Seeds/plant (flowering only) |  |  |  |  |
|  | LH | Seed per plant |  |  |  |  |
| Hall et al. (2010) | M | Maximun rossette diameter | Herb | Scrophulariaceae | † | Coastal |
|  | M | Plant height |  |  |  |  |
| Helenurm (1998) | LH | Fruit production per plant | Herb | Fabaceae | Annual | Desert |
| Hereford and Moriuchi (2005) | LH | Seed weight | Herb | Rubiaceae | Annual | Old field |
|  | LH | Proportion germinating |  |  |  |  |
| Heschel et al.( 2002) | P | Carbon assimilation rate (A) | Herb | Balsaminaceae | Annual | Old field |
|  | P | Stomatal conductance (g) |  |  |  |  |
|  | P | Water-use efficiency (WUE) |  |  |  |  |
| Jakobsson and Dinnetz (2005) | M | Log rossette size | Herb | Asteraceae | Perennial | Old field |
|  | M | Leaf width |  |  |  |  |
|  | M | Teeth per leaf |  |  |  |  |
|  | M | Plant height |  |  |  |  |
|  | M | Internode length |  |  |  |  |
|  | M | Nodes per second branch |  |  |  |  |
|  | M | Length of longest leaf |  |  |  |  |
| Jordan (1992) | LH | Seeds/plants 1983 | Herb | Rubiaceae | Annual | Old field |
|  | LH | Survival |  |  |  |  |
| Knight and Miller (2004) | M | Number of Leaves | Herb | Araliaceae | Perennial | Coastal |
|  | M | Internode length |  |  |  |  |
|  | M | Petiole length |  |  |  |  |
|  | M | Leaf width |  |  |  |  |
|  | M | Mean biomass |  |  |  |  |
| Lowry and Willis (2010) | LH | Days to flowering | Herb | Scrophulariaceae | † | Coastal |
|  | LH | Flower produced |  |  |  |  |
| Miller and Weis (1999) | M | Leaf length | Shrub | Asteraceae | Perennial | Coastal |
| Platenkamp (1990) | LH | Mean number of inflorescences | Grass | Poaceae | Perennial | Grassland |
| Radford and Cousens (2000) | LH | Survival percentage | Herb | Asteraceae | † | Coastal |
| Rice and Mack (1991) | LH | Percentage of survival to reproduction | Grass | Poaceae | Annual | Desert |
|  | M | Individual plant dry weight |  |  |  |  |
|  | LH | Average No. of seed per plant |  |  |  |  |
|  | LH | Net reproductive rate |  |  |  |  |
| Richards et al. (2011) | P | WUE | Shrub | Asteraceae | Perennial | Coastal |
|  | P | Leaf calcium |  |  |  |  |
|  | M | Final height |  |  |  |  |
|  | P | Leaf phosphorous |  |  |  |  |
|  | P | Leaf Nitrogen |  |  |  |  |
|  | P | Leaf Potassium |  |  |  |  |
|  | M | Leaf size |  |  |  |  |
|  | M | Total leaves |  |  |  |  |
|  | P | Leave Sodium |  |  |  |  |
|  | P | Leaf Magnesium |  |  |  |  |
|  | M | Succulence |  |  |  |  |
|  | M | Total biomass |  |  |  |  |
| Scheiner and Teeri (1986) | M | No. of culms | Herb | Poaceae | Perennial | Old field |
|  | M | Length longest vegetative leaves |  |  |  |  |
|  | LH | No.of flowering stalks |  |  |  |  |
|  | LH | Mean No. flowering stalk length |  |  |  |  |
|  | LH | Mean No. of spikelets |  |  |  |  |
|  | M | Flag leaf length |  |  |  |  |
|  | LH | No. of days to spikelet emergence |  |  |  |  |
|  | LH | No. of days from spikelet emergence to seed release |  |  |  |  |
|  | M | Percentage of aboveground biomass as flowering stalks |  |  |  |  |
|  | M | Total plant dry weight |  |  |  |  |
| Stanton and Galen (1997) | M | Leaf number | Herb | Polemoniaceae | Annual | Alpine tundra |
|  | M | Leaf length |  | Ranunculaceae | Perennial |  |
| Verhoeven et al. (2004) | LH | Viability | Grass | Poaceae | Perennial | Coastal |
|  | LH | Heads/plant |  |  |  |  |
|  | LH | Seeds/head |  |  |  |  |
|  | LH | Seed weight |  |  |  |  |
| Volis et al. (2002) | LH | Fecundity | Herb | Ranunculaceae | Perennial | Desert |
|  | LH | Survival of seedling | Grass | Poaceae | Annual |  |
| Wang et al. (1997) | LH | No. of inflorescences per flowering | Shrub | Asteraceae | Perennial | Desert |
|  | LH | Average total inflorescence length |  |  |  |  |

**Table S2**

| **A. Records by pair** | **N** | Not plastic | Plastic |  |  |  |
| --- | --- | --- | --- | --- | --- | --- |
|  | 362 | 44.2 | 55.8 |  |  |  |
|  |  |  |  |  |  |  |
| **B. Records by block** | **N** | Not plastic | Plastic |  |  |  |
|  | 181 | 33.7 | 66.3 |  |  |  |
|  |  |  |  | *Adaptive Plasticity* | |  |
|  | **N** | **Pattern 1: Canalization**  **No differentiation** | **Pattern 2:**  **Canalization Population differentiation** | **Pattern 3: Perfect Adaptive Plasticity** | **Pattern 4: Adaptive Plasticity** | **Pattern 5: Non adaptive Plasticity** |
| All | 181 | 6.6 | 27.1 | 21.5 | 24.3 | 20.4 |
| Life history | 92 | 5.4 | 29.3 | 18.5 | 22.8 | 23.9 |
| Morphological | 79 | 6.3 | 22.8 | 25.3 | 27.8 | 17.7 |
| Physiological | 10 | 20 | 40 | 20 | 10 | 10 |

**Table S3**

| **A. Records by pair** | **N** | Not plastic | Plastic |  |  |  |
| --- | --- | --- | --- | --- | --- | --- |
|  | 362 | 91.2 | 8.8 |  |  |  |
|  |  |  |  |  |  |  |
| **B. Records by block** | **N** | Not plastic | Plastic |  |  |  |
|  | 181 | 83.4 | 16.6 |  |  |  |
|  |  |  |  | *Adaptive Plasticity* | |  |
|  | **N** | **Pattern 1: Canalization**  **No differentiation** | **Pattern 2:**  **Canalization Population differentiation** | **Pattern 3: Perfect Adaptive Plasticity** | **Pattern 4: Adaptive Plasticity** | **Pattern 5: Non adaptive Plasticity** |
| All | 181 | 19.9 | 63.5 | 13.5 | 0 | 3 |
| Life history | 92 | 21.7 | 58.7 | 16.8 | 0 | 2.7 |
| Morphological | 79 | 16.5 | 68.4 | 11.4 | 0 | 3.8 |
| Physiological | 10 | 30 | 70 | 0 | 0 | 0 |
